# 2-Dodecyl-6-methoxycyclohexa-2,5-dien-1,4-dione alleviates liver fibrosis and improves intestinal flora and bile acid metabolism

**DOI:** 10.3389/fphar.2025.1581138

**Published:** 2025-05-16

**Authors:** Yongfei He, Thi Thai Hoa Pham, Junming Xu, Shengjie Hong, Jicai Wang, Hang Zhai, Qiang Tao, Ruixi Li, Guangquan Zhang, Xianjie Shi

**Affiliations:** ^1^ Department of Hepatobiliary and Pancreatic Surgery, The Eighth Affiliated Hospital of Sun Yat-sen University, Shenzhen, Guangdong, China; ^2^ Zhuang & Yao Medicine Research and Development Center, Guangxi International Zhuang Medicine Hospital is Affiliated to Guangxi University of Chinese Medicine, Nanning, Guangxi Zhuang, China

**Keywords:** 2-dodecyl-6-methoxycyclohexa-2,5-dien-1,4-dione, liver fibrosis, bile acid metabolism, intestinal flora, transcriptomics

## Abstract

**Background:**

The bioactive compound 2-Dodecyl-6-methoxycyclohexa-2,5-diene-1,4-dione (DMDD), derived from the horn root of star fruit, exhibits therapeutic promise through its modulation of the TGF-β1 pathway and regulation of bile acids.

**Methods:**

In this study, a liver fibrosis model was established in Kunming mice (KM) induced by carbon tetrachloride (CCL4), and DMDD (50 mg/kg) was administered intragastrically. HE staining, Masson staining, and Sirius staining were used to evaluate the effect of DMDD on liver fibrosis. The Illumina sequencing platform was used to detect intestinal flora and liver transcriptome information in mouse feces, and high-performance liquid chromatography-tandem mass spectrometry (HPLC-MS/MS) technology was used to detect bile acid content changes in mouse feces.

**Results:**

The results show that DMDD can mitigate liver fibrosis-induced damage in mice, potentially through the suppression of the TGF-β/Smad signaling pathway. Furthermore, DMDD increased the abundance of *Lactobacillus*, *Bacteroides*, *Ruminococcaceae*, *Ruminococcus*, and *Oscillospira*, thereby addressing intestinal flora disturbances and regulating bile acid metabolism.

**Conclusion:**

Our study suggests that DMDD alleviates liver fibrosis by inhibiting the TGF-β/Smad signaling pathway, restoring gut microbiota homeostasis, and balancing bile acid metabolism.

## Introduction

Liver fibrosis, that is, excessive deposition of the extracellular matrix (ECM) in the liver, is essentially a repair mechanism for chronic liver injury. However, this repair process may lead to a series of adverse consequences such as liver cirrhosis, portal hypertension, liver failure, and even an increased risk of liver cancer (Tiasha and Venkatraman, 2023). Epidemiological data show that approximately 2 million patients worldwide die from end-stage liver disease caused by liver fibrosis every year, which poses a heavy burden on personal health and social economy (Ali et al., 2014; Fu-Sheng et al., 2014). Currently, there are no clinically approved effective antifibrotic therapies for slowing or reversing liver fibrosis. Targeted therapy is the most effective method to prevent and treat liver fibrosis. Although liver fibrosis can be slowed or reversed by eliminating pathogens, some etiologies cannot be eliminated or eliminated too slowly to prevent the progression of liver fibrosis and life-threatening complications (Ginès et al., 2021). Drug intervention in the treatment of liver fibrosis is the main means at present, and some anti-fibrosis drugs have been proven to have the potential function of inhibiting the progression of anti-fibrosis in animal studies. However, most drugs for the treatment of liver fibrosis, such as interferons, PPARγ agonists, antioxidants, and anti-inflammatory drugs, are still in the clinical development stage (Maurizio and Massimo, 2023). Therefore, the development of safe and effective antifibrotic drugs is urgently required.

In the face of the challenge of liver fibrosis treatment, traditional Chinese medicine shows unique advantages. Studies have shown that a variety of traditional Chinese medicine monomer compounds, such as puerarin, silymarin, umbelliferone, tanshinone, emodin, and curcumin, can effectively act on hepatic stellate cells (HSCs), thus playing an important role in the treatment of liver fibrosis (Asmaa et al., 2021; Yanwei et al., 2023; Yongxiang et al., 2023). With the characteristics of “multi-component, multi-target, and multi-channel”, these traditional Chinese medicines have not only performed well in promoting the reversal of liver fibrosis but also achieved remarkable results in inhibiting the progression of liver fibrosis. Whether viral, cholestatic, or alcohol-induced liver fibrosis, traditional Chinese medicine has shown good therapeutic effects. Verrhoa carambola L (0xalidaceae) Root (ACLR) is a plant belonging to the family of the pressing plant. It is mainly used in traditional Chinese medicine to treat diseases, such as diabetes, urolithiasis, joint pain, and chronic paroxymorial headache. Our team has previously extracted the monomer compound 2-dodecyl-6-methoxycyclohexa-2,5-diene-1.4-dione (DMDD) from ACLR. Previously, we successfully isolated a monomeric compound named 2-dodecyl-6-methoxycyclohexa-2,5-diene-1,4-dione (DMDD) from Averrhoa carambola L (ACLR). Subsequently, we verified that DMDD exhibited significant inhibitory effects on tumor cells in various tumor types, including cholangiocarcinoma, breast cancer, and lung cancer (Chunxia et al., 2017; Yongfei et al., 2022). DMDD not only excels in tumor treatment but also plays an active role in improving chronic non-tumor diseases such as diabetes and insulin resistance. Additionally, it is effective in delaying the progression of diabetic kidney disease and provides new hope for the treatment of related diseases (Hongliang et al., 2020). However, the role of DMDD in liver fibrosis and its underlying mechanisms remain unknown and need to be clarified.

Owing to the anatomical relationship between the liver and intestine, as well as the enterohepatic circulation mechanism of bile acid metabolism, the impact of gut microbiota metabolites on the liver is crucial in the etiology of liver diseases. Many traditional Chinese herbal medicines and monomers have been proven to protect the liver by regulating the intestinal flora, inhibiting intestinal barrier dysfunction, and modulating bile acid metabolism through the gut-liver axis. The gut-liver axis is an important focus in the treatment of liver fibrosis. In this study, we established a mouse model of liver fibrosis using carbon tetrachloride (CCL4) to evaluate the effects of DMDD and further explored the potential mechanism of action and its effects on intestinal flora and bile acid metabolism, providing a basis for the development of anti-liver fibrosis drugs.

## Materials and methods

### Extraction and purification of DMDD

The star fruit root was collected from Lingshan County, Qinzhou City, Guangxi Zhuang Autonomous Region, and was identified by Professor Lai Maoxiang of the Guangxi Academy of Traditional Chinese Medicine (CN112315987A). The star fruit root was dried, and 18 kg of crushed material was passed through a 10-20 mesh sieve to obtain the appropriate particle size of the medicinal powder. The screened medicinal material powder was added into a certain volume of ethanol-water mixture (144 kg, including 86.4 kg ethanol and 57.6 kg water), thoroughly mixed, and left to soak for 30 min so that the medicinal material components were fully dissolved in the solvent. Then, under atmospheric pressure, reflux extraction was carried out for 1.5 h to ensure that the active ingredients in the medicinal materials were fully extracted. After the extraction was completed, the filtration operation was carried out, and the extraction was repeated three times, each time adding a 144 kg ethanol-water mixture and then combining the filtrate obtained three times to obtain the alcohol extract of star fruit root. The resulting alcohol extract was finally concentrated at a reduced pressure of 60°C to remove the solvent and concentrated to about 9 L. Then, the concentrated alcohol extract of star fruit root was extracted with cyclohexane three times, each time using three times the amount of cyclohexane, and combined with three times the extraction liquid. Then, different proportions of cyclohexane-ethyl acetate solution (the proportions are 100:0, 20:1, 18:1, 15:1, 12:1, 10:1, 8:1, 5:1, 3:1, 1:1, 0:100) were used to eluate the extract to separate different components. The residues were detected by TLC with ethyl acetoacetate-methanol 1:1 as the development agent, and the same fractions were combined. After elution, repeated recrystallization of precipitated crystallization with methanol was performed to further purify the compound. Finally, the monomer compound DMDD (2-Dodecy1-6-methoxycyclohexa-2, 5-Dien-1,4-dione) was obtained by high-performance liquid chromatography (HPLC) with a purity of 95%.

### Animals

SPF male KM mice aged 3–4 weeks were selected for the experiment. The mice were purchased from Shenzhen TOPBIOTH with license number SYXK (Guangdong) 2020–0230. They were housed in an animal room at a temperature of (22 ± 2) degree centigrade, humidity of 50%–60%, and no special pathogens, and were free to consume water and feed. After 1 week of adaptive feeding, a total of 12 mice were randomly divided into 3 groups (CON group, MOD group, DMDD group) with 4 mice in each group. The use of this small sample size was based on an initial exploratory study designed to minimize animal use while ensuring statistical validity, and subsequent studies will expand the sample size for validation. The dose of DMDD (50 mg/kg) was determined based on previous pharmacokinetic studies that showed efficacy and safety in mouse models^11^. A single dose was used to establish a proof of concept, and subsequent dose-response studies are planned. The model and treatment groups were subcutaneous injected with a mixture of CCL4 and olive oil (CCL4: olive oil = 1:7) at 2 mL/kg, and the model group was subcutaneous injected with olive oil at 2 mL/kg, twice a week. After the third week of modeling, the DMDD group was administered DMDD (50 mg/kg) by gavage every other day, while the control and model groups were administered the same amount of normal saline by gavage. The weight changes of mice were recorded every week for 3 weeks, and none of the mice experienced adverse reactions or died during treatment. Body weight changes during the experiment were recorded in Supplementary Table S1.

### Liver function test

Blood was collected from the abdominal aorta and centrifuged at 13,000 r/min for 10 min, and the supernatant was collected. Serum alanine aminotransferase (ALT) and aspartate aminotransferase (AST) levels were measured using a Mindray bs-420 automatic biochemical instrument (Shenzhen Mindray Biomedical Electronics Co. Ltd.).

### Histological stain

The liver and small intestine tissues were fixed with 4% paraformaldehyde, dehydrated with fractional ethanol, transparent with xylene, embedded in paraffin, and cut into 5 μM thick tissue sections. The liver was stained with HE, Masson, and Sirius red staining kits, and the small intestine was stained with HE.

### Transcriptome sequencing

Liver tissue was weighed and RNA was extracted according to the operation of the total RNA kit I (r6834-01, omega). Subsequently, RNA purity and concentration were detected, mRNA with a Polya tail was enriched by oligo (DT) magnetic beads, and the resulting mRNA was randomly interrupted by divalent cations in the NEB fragmentation buffer. Using fragmented mRNA as a template and random oligonucleotides as primers, the first strand of cDNA was synthesized in the M-MuLV reverse transcriptase system, followed by RNA strand degradation with RNase H. The second strand of cDNA was synthesized from dNTPs in a DNA polymerase I system. Purified double-stranded cDNA was subjected to end repair, end repair, a-tailing, and sequencing adaptor ligation. The cDNA of approximately 250-300 BP was screened with impure XP beads, PCR amplification was performed, the PCR product was purified again with impure XP beads, and finally, the library was obtained. After library construction, transcriptome sequencing was performed using the Illumina HiSeq platform.

### RT-qPCR

The liver tissue was weighed, RNA was extracted according to the above method, and total RNA was reverse transcribed into cDNA using a reverse transcription kit. Actin was used as an internal reference gene, with three replicates for each gene in each group. The target gene was amplified according to the instructions of the RT-PCR Expansion Kit and the relative expression of each mRNA was calculated. Primer sequences are shown in Table 1.

**TABLE 1:** List primers for RT-qPCR.

Gene(Mouse)	Forward primer (5′–3′)	Reverse primer (5′–3′)
β-ACTIN	GCC​GGA​CTC​ATC​GTA​CTC​C	GTG​ACG​TTG​ACA​TCC​GTA​AAG​A
α-SMA	AGA​CCT​TCA​ATG​TCC​CTG​CCA	GTT​GTG​AGT​CAC​GCC​ATC​TCC
COL1A1	TGC​TAA​CGT​GGT​TCG​TGA​CCG​T	ACA​TCT​TGA​GGT​CGC​GGC​ATG​T
TIMP1	GAG​ACC​ACC​TTA​TAC​CAG​CGT​T	TAC​GCC​AGG​GAA​CCA​AGA​AG
TGF-β	ATG​GTG​GAC​CGC​AAC​AAC​GC	GGC​ACT​GCT​TCC​CGA​ATG​TCT​G
SMAD2	CCG​TGC​TCC​CTC​CGT​CTT​CC	CTGCCGCCCGCTGATTGG
SMAD3	GTT​GCC​TGA​AGC​CTG​GAA​GTG​G	TCC​TGC​CGT​CTG​TTG​AAT​GTG​C
CYP7A1	GAC​CAA​GTC​TTT​CCG​GCA​C	CAG​AGA​ATA​GCG​AGG​TGC​GT
CYP8B1	TCC​CCT​ATC​TCT​CAG​TAC​ACA​T	ACT​TGT​AGA​AGT​CCA​CTT​TCC​G
CYP27A1	AGA​CCA​TCG​GCA​CCT​TTC​CTG​AG	GCA​CCA​CAC​CAG​TCA​CTT​CCT​TG
NTCP	CAA​ACC​TCA​GAA​GGA​CCA​AAC​A	GTA​GGA​GGA​TTA​TTC​CCG​TTG​TG
BSEP	TCT​GAC​TCA​GTG​ATT​CTT​CGC​A	CCC​ATA​AAC​ATC​AGC​CAG​TTG​T
MRP2	GTG​TGG​ATT​CCC​TTG​GGC​TTT	CAC​AAC​GAA​CAC​CTG​CTT​GG

### Specimen collection

After the last intervention, mice in each group were fasted overnight and were given 1.25% avodin solution (0.02 mL per 10 g of body weight) for intraperitoneal anesthesia, and blood was taken from the abdominal aorta to detect relevant indicators. The liver tissues of the mice were separated and weighed, and the liver index = (liver wet weight ÷ body mass) × 100%. Part of the liver tissues was fixed in 4% paraformaldehyde for histopathological observation, and the rest of the liver tissues were stored in at −80°C refrigerator. 0.2 g of cecum contents of mice in each group were collected and placed in a sterile centrifuge tube and stored in a −80°C refrigerator. Small intestine tissues were fixed in 4% paraformaldehyde for histopathological observation.

### Microbiological analysis

The total genomic DNA of the samples was extracted using the CTAB method. The DNA concentration and purity were assessed on a 1% agarose gel. Subsequently, the DNA was diluted to 1 ng/μL with sterile water based on the concentration to meet the requirements of subsequent experiments. For the microbiological analysis, primers 341F (5′-CCTAYGGGRBGCASCAG-3′) and 806R (5′-GGACTACNNGGGTATCTAAT-3′) were used to amplify the V3-V4 regions of bacterial 16S rRNA genes. The PCR products were then recovered using a universal DNA purification recovery kit (Tiangen, China). Library construction was carried out using the NEB Next^®^ Ultra DNA library prep kit. The constructed library was detected and quantified by Q-PCR using Agilent 5,400. Once the library met the quality standards, 16S rRNA gene sequencing targeting the V3-V4 hypervariable regions was performed using the Illumina HiSeq platform for online sequencing.

Raw sequencing data were processed using QIIME2 (version 2020.2). Sequences were filtered, denoised, and clustered into amplicon sequence variants (ASVs) via the DADA2 pipeline. Taxonomic classification was performed using the SILVA reference database (v138, https://www.arb-silva.de/). LefSe and other methods were employed to identify bacteria with differences in abundance between groups and samples using PICRUSt2 (https://github.com/picrust/picrust2/wiki). KEGG pathway functional analysis was conducted on the species sequences. Differences in function were analyzed using STAMP version 2.1.3 statistical software (http://kiwi.cs.dal.ca/Software/STAMP).

In summary, this study utilized a comprehensive approach starting from DNA extraction, through targeted amplification of specific 16S rRNA gene regions, library construction, high-throughput sequencing, and finally, detailed bioinformatics analysis to investigate the intestinal flora and its functional characteristics.

### Bile acid sequencing

Samples were extracted in 400 μL of methanol(−20°C centigrade)with two steel balls, vortexed for 60 s. Put in a tissue grinder, ground at 55 Hz for 1 min, and repeat the above operation at least twice. Sonicate for 30 min at room temperature, centrifuged at 12,000 rpm and 4°C centigrade for 10 min, take 300 μL of the supernatant was taken, 600 μL of water to mix, and vortexed for 30 s. Then, an appropriate amount of supernatant was add 30% methanol to dilute 5 times. The supernatant was filtered through a 0.22 μm membrane, and the filtrate was added to the LC-MS bottle. LC analysis was performed using EXion LC Liquid chromatography (AB SCIEX, United States). The mass spectrometric detection of metabolites was performed using AB6500 Plus (AB SCIEX, United States).

### Western blot assay

The liver tissues from the aforementioned mouse were collected, and RIPA buffer was added to achieve complete protein lysis. Subsequently, the supernatant, which comprised the total tissue protein, was isolated using a pre-cooled ultra-high-speed centrifuge at a temperature of 4°C. The concentration of protein was measured using a BCA assay kit. Following this, SDS-PAGE electrophoresis and membrane transfer were conducted. The primary and secondary antibodies were then diluted as per the manufacturer’s guidelines. Lastly, ECL development was performed. TGF beta 1 Polyclonal antibody (Cat No. 26155-1-AP, Proteintech), TGFB2/TGF-beta 2 Polyclonal antibody (Cat No. 28426-1-AP, Proteintech), TGF Beta 3 Polyclonal antibody (Cat No. 18942-1-AP, Proteintech), SMAD2/SMAD3 Rabbit pAb (Cat No. A18674, ABclonal), Phospho-Smad3-T179 Rabbit pAb (Cat No. AP0554, ABclonal), Alpha smooth muscle actin Polyclonal antibody (Cat No. 23081-1-AP, Proteintech), Collagen Type I Monoclonal antibody (Cat No. 67288-1-Ig, Proteintech), Vinculin Rabbit mAb (Cat No. A2752, Abclonal), HRP Goat Anti-Rabbit IgG (Cat No. AS014, Abclonal), HRP Goat Anti-Rabbit IgG (Cat No. AS003, Abclonal). ZO-1 Polyclonal antibody (Cat No. 21773-1-AP, Proteintech), Occludin Polyclonal antibody (Cat No. 27260-1-AP, Proteintech).

### Statistical analysis

Statistical analyses were performed using SPSS 22.0. The results of three repeated measurements are expressed as mean ± standard deviation (SD), and the values were statistically compared using the t-test or rank sum test. Differences of the intestinal flora and bile acid in oebiotech (https://cloud.oebiotech.com) for correlation analysis.

The edgeR software was used to analyze the number of gene expression differences among the groups, and FDR and log 2 FC genetic variants were used for screening. The screening conditions were FDR ≤0.05, |log2foldchange| >1. For GO and enrichment analyses of differential genes, cluster analysis software was used to perform go and do functional enrichment analysis of differential genes and KEGG pathway enrichment analysis. FDR ≤0.01 was used as the threshold of signal pathway gene function and enrichment analysis to detect and correct LPBM. Statistical significance was set at P < 0.05.

## Results

### DMDD alleviates CCL4-induced liver fibrosis in mice

**FIGURE 1:**
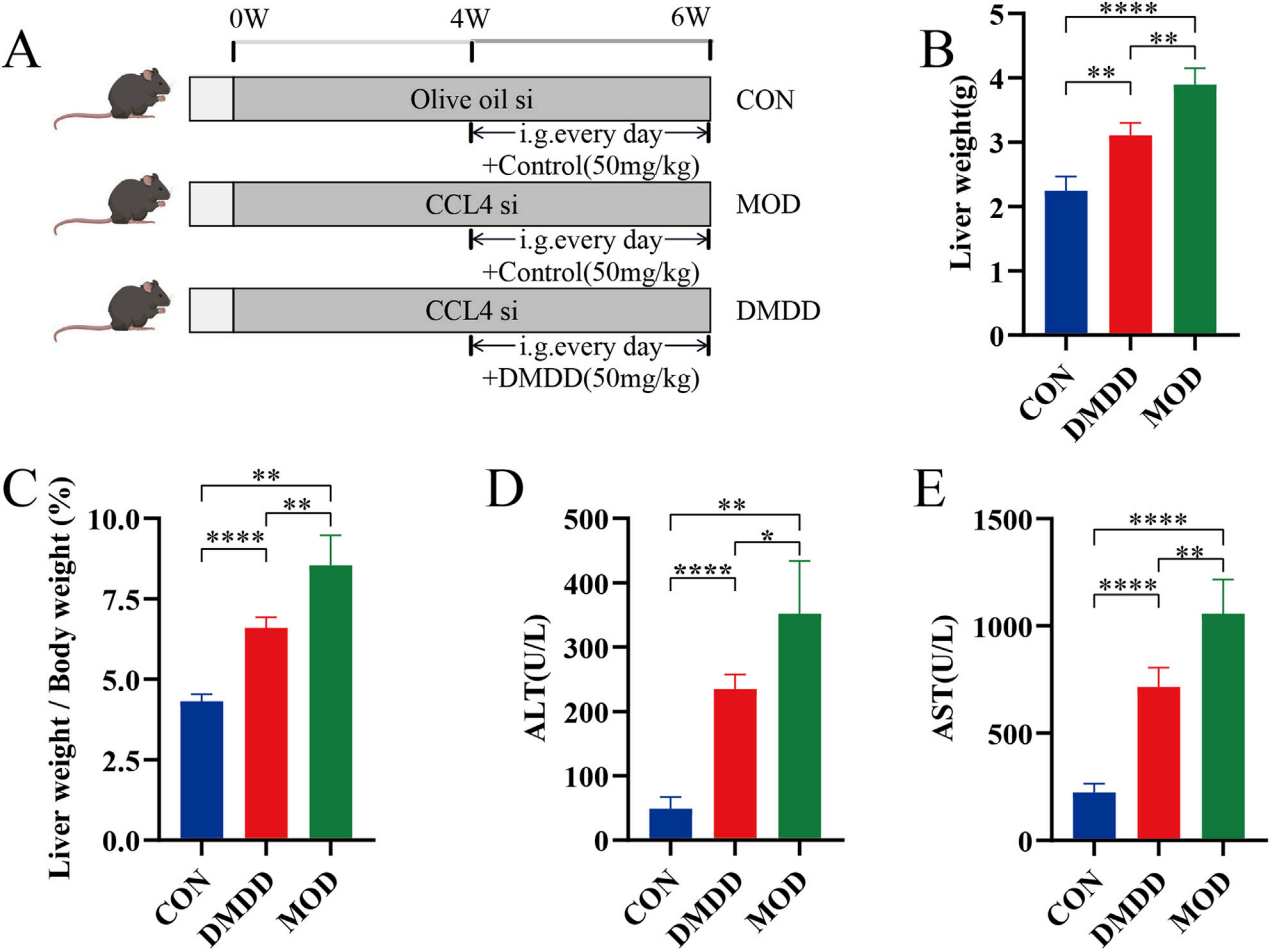
DMDD alleviates CCL4-induced liver injury in mice. **(A)** Schematic diagram of DMDD in the treatment of CCL4-induced liver fibrosis. Starting from week 4, the patients were given daily administration of normal saline or DMDD (50 mg/kg) for 3 consecutive weeks, with n = 4 per group. **(B)** Liver weight of each group of mice. **(C)** Liver accounted for 100% of body weight in each group. **(D, E)** Serum ALT and AST of each group of mice. si subcutaneous injection. *P < 0.05, **P < 0.01,***P < 0.001,****P < 0.0001.

**FIGURE 2:**
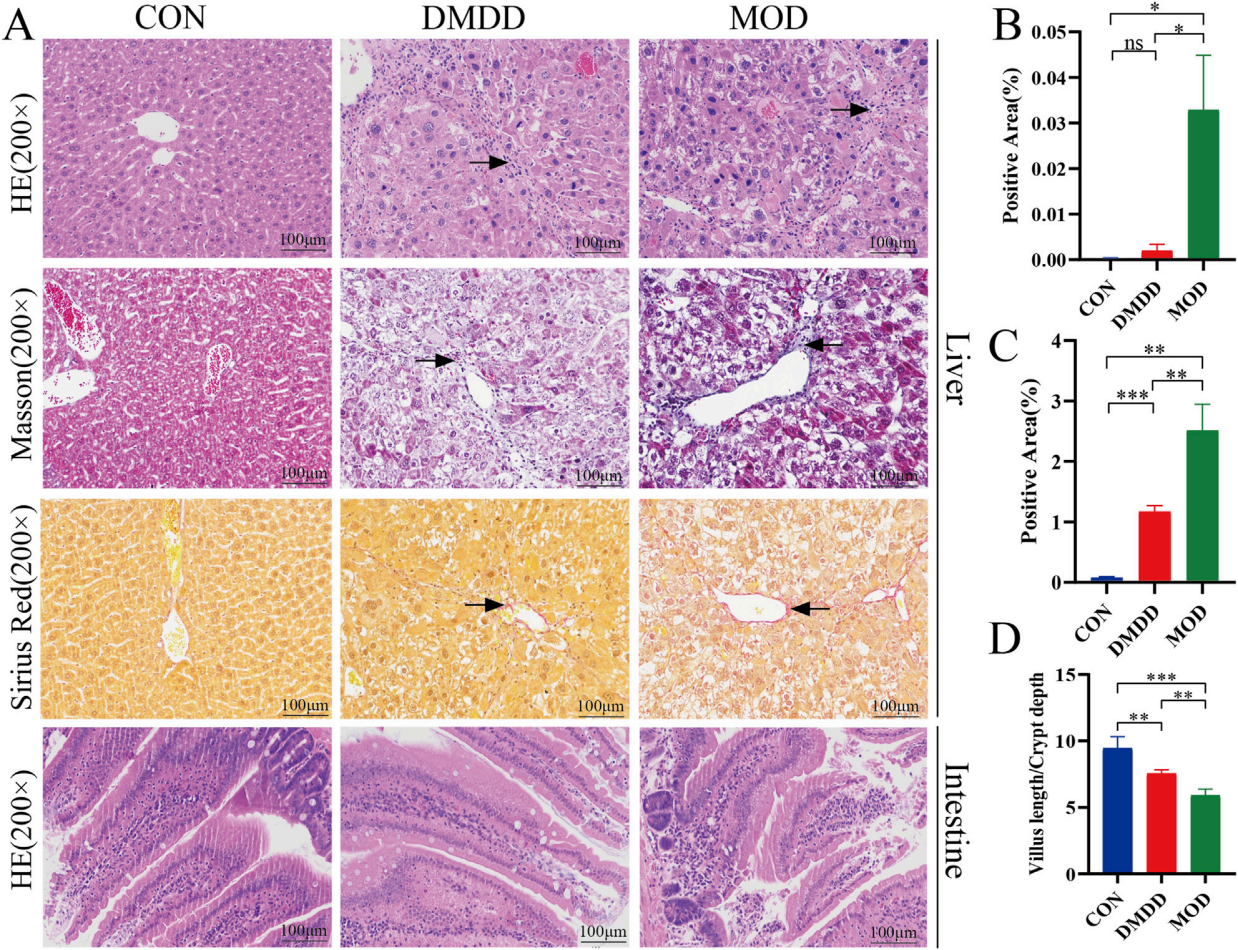
DMDD alleviates CCL4-induced liver fibrosis in mice. **(A)** Liver HE, Masson and Sirius red staining, and small intestine HE staining from top to bottom, respectively. **(B, C)** Are the percentages of Masson and Sirius red-positive cells in the liver, respectively. **(D)** Is the depth of the small intestinal villi/lacunae. The black arrows show areas of fibrosis and/or hyperplasia. Scale bar, 100 μm*P < 0.05, **P < 0.01,***P < 0.001,****P < 0.0001.

Although DMDD has been shown to be effective in various disease mouse models, to understand whether DMDD can alleviate liver fibrosis, we adopted the most commonly used CCL4-induced method to construct a mouse model of liver fibrosis while applying DMDD for treatment (Figure 1A). At the end of the experiment, the mice were dissected and their livers were weighed. It was found that the DMDD treatment group reduced the weight of the liver with CCL4-induced liver fibrosis (Figure 1B). To further quantitatively evaluate the effect of DMDD on the liver, we calculated the percentage of liver weight, suggesting that DMDD can alleviate acute liver swelling caused by CCL4 (Figure 1C). To further explore the effect of DMDD on liver function, the serum of mice was used to measure ALT and AST levels. The results showed that the ALT and AST levels of mice in the DMDD treatment group were significantly decreased (Figure 1D,E). Histological staining showed that the connective tissue around the central vein and the portal area of the liver in the CCL4 model group proliferated, accompanied by inflammatory cell infiltration, and fiber bridging was formed at the same time. However, in the DMDD group, only a fibrous septum was formed, accompanied by a small amount of inflammatory cell infiltration, and liver injury was reduced (Figures 2A,B). To demonstrate the changes in extracellular matrix collagen, Masson staining and Sirius red staining were used to observe collagen accumulation. There was no collagen production in the normal control group, a large amount of collagen production in the model group, and decreased collagen production in the DMDD group (Figures 2A–C).

### DMDD alleviates intestinal barrier damage in mice with liver fibrosis

Intestinal barrier function is crucial for maintaining liver health. When intestinal barrier function is impaired, harmful substances in the intestine may enter the blood circulation and affect the liver, leading to the occurrence of liver diseases such as liver fibrosis. To understand changes in the intestine, we performed histological staining of the small intestine of mice. Villus length (V) and crypt depth (C) are important indicators of small-intestinal growth and nutrient absorption. The V/C ratio reflects the functional status of small intestine. A decrease in the ratio indicated mucosal damage and reduced nutrient absorption. It can be seen that in the model group, a large number of intestinal villi were edematous, the gap between the epithelium and the lamina propria was widened, a large amount of intestinal epithelial cells were shed, the lamina propria was exposed, and there were few goblet cells. At the same time, the ratio of villus height to crypt depth was reduced. Although the villi of the intestinal tissue were edematous, there was no significant intestinal epithelial cell shedding and lamina propria denudation, and the ratio of villus height to crypt depth was improved compared with the model group (Figures 2A–D). DMDD treatment restored the expression of intestinal tight junction proteins ZO-1 and Occludin, as evidenced by Western blot (Supplementary Figure S2), indicating improved intestinal barrier integrity. The above results showed that DMDD could improve intestinal barrier function damage caused by liver fibers.

### DMDD improves liver fibrosis in mice through TGF-β signaling pathway

**FIGURE 3:**
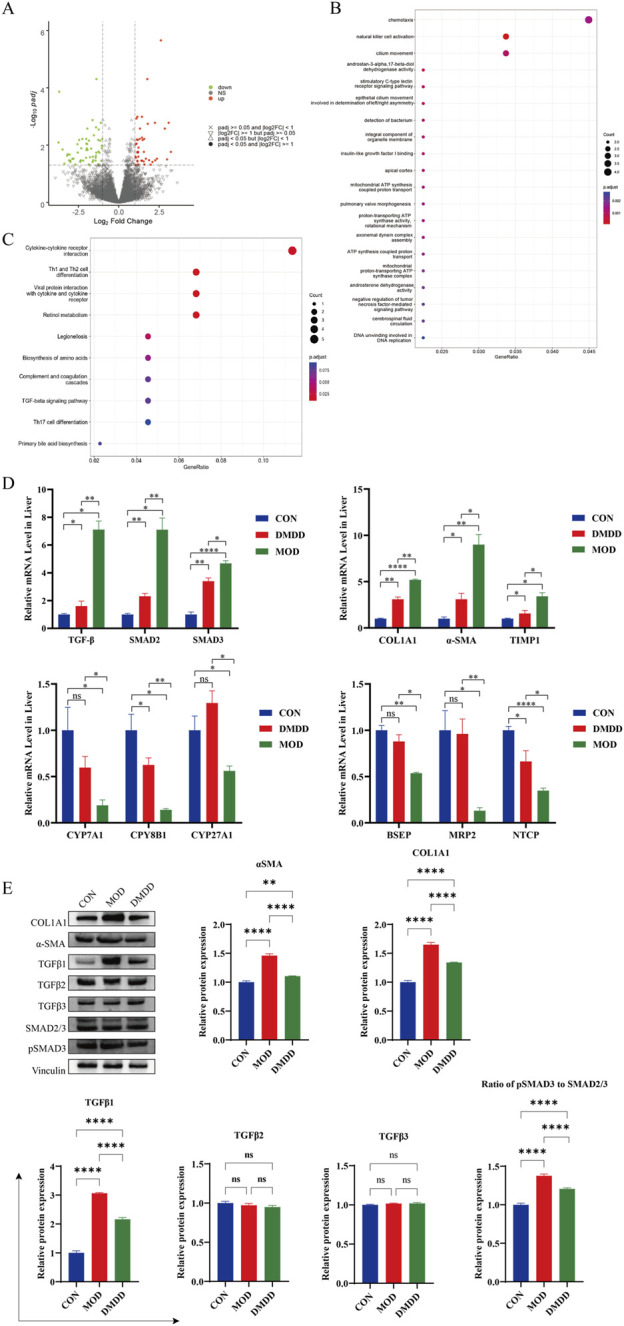
Transcriptome sequencing analysis. Transcriptome sequencing was performed on liver tissues of CCL4 model group and DMDD treatment group, and differential expression and enrichment analysis were performed. **(A)** Volcano map of differentially expressed genes. **(B)** Differential expression gene GO analysis, FDR value ranked top 20. **(C)** KEGG pathway enrichment analysis of differentially expressed genes, FDR value ranked top 10. **(D)** mRNA expression levels of TGF-β/Smad signaling pathway, liver fibrosis and key genes in bile acid metabolism. **(E)** Expression of key proteins of TGF-β/Smad pathway and fibrotic proteins.*P < 0.05, **P < 0.01,***P < 0.001,****P < 0.0001. ns, No significant difference.

To identify the signaling pathways involved in DMDD alleviation of liver fibrosis, we performed transcriptome sequencing of the CCL4 model group and DMDD-treated mouse liver. There were a large number of differentially expressed genes in the CCL4 model group compared to the DMDD treatment group. GO analysis suggested that it was related to chemotaxis and ATP synthesis, coupled with proton transport and other functions. KEGG enrichment analysis showed that there were significant differences in cytokine receptors; Th1, Th2, and Th17 cell differentiation; and TGF-β and other signaling pathways (Figure 3A–C). Therefore, we verified the mRNA expression levels of the TGF-β/Smad2/SMAD3 pathway, liver fibrosis, and bile acid metabolism genes. Compared with the MOD group, the TGF-β/Smad2/SMAD3 pathway was significantly inhibited after DMDD intervention, and liver fibrosis indices COL1A1, α-SMA, and TIMP1 were significantly downregulated, while bile acid metabolism of CYP7A1, CPY8B1, CYP27A1, BSEP, MRP2, and NTCP were significantly upregulated (Figure 3D). The results of Western blot analysis indicate that DMDD exerts a selective effect on the expression of TGF-β family members, significantly reducing the expression level of TGF-β1. At the same time, no significant changes were observed in the expression of TGF-β2 and TGF-β3. Additionally, DMDD specifically regulates the TGF-β/Smad2/3 signaling pathway by blocking the phosphorylation process of Smad3, without having a notable impact on other TGF-β subtypes. Further analysis confirms that DMDD also significantly decreases the protein expression levels of α-SMA and COL1A1, consistent with their changes at the mRNA level (Figure 3E). These results suggest that DMDD inhibits the TGF-β/Smad2/Smad3 pathway and plays a role in inhibiting the progression of liver fibrosis.

### DMDD regulates intestinal flora in mice with liver fibrosis

**FIGURE 4:**
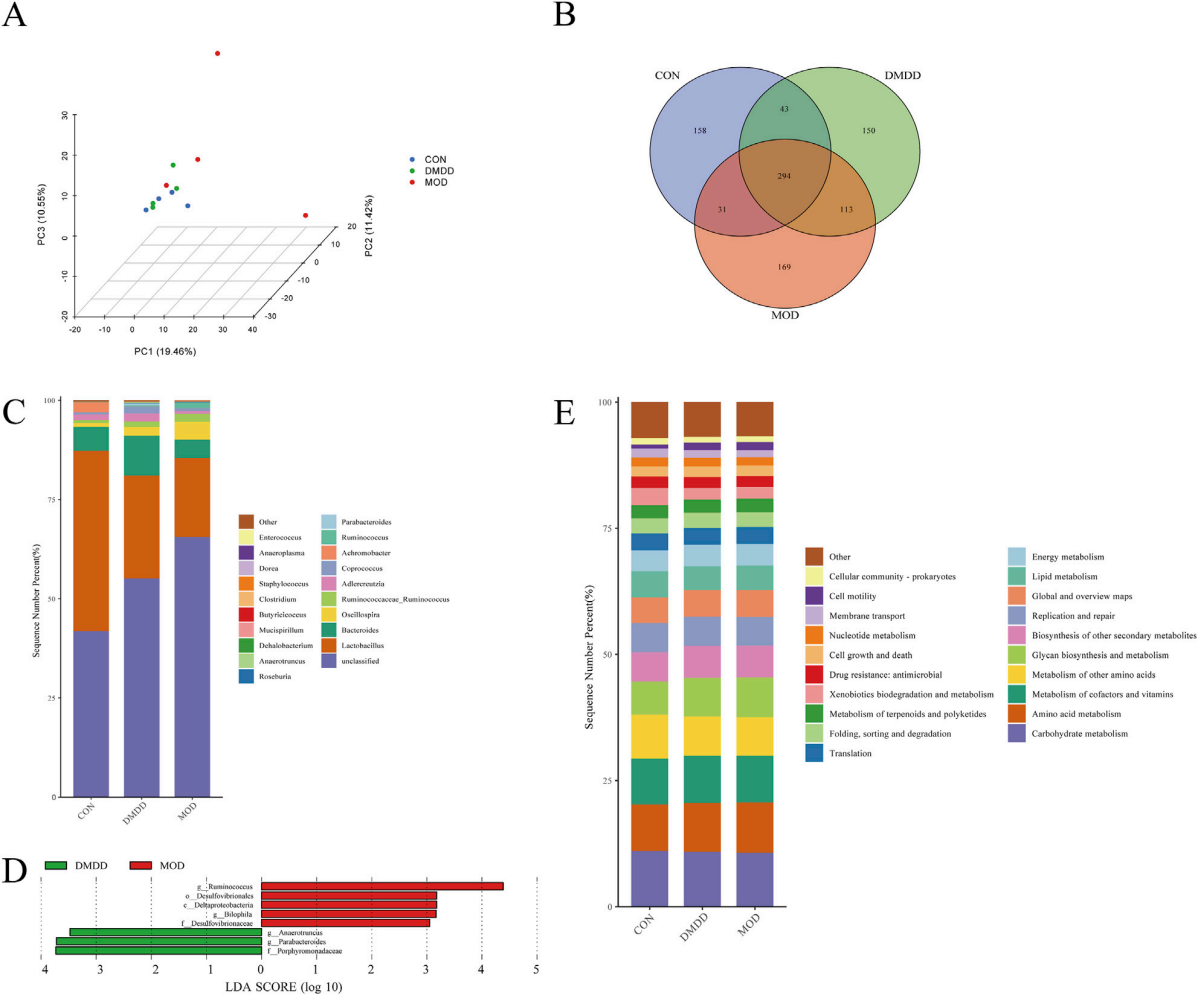
DMDD improves intestinal microbiota in CCL4-induced liver fibrosis mice. **(A)** Principal Component Analysis (PCA) of CON, MOD, and DMDD groups. **(B)** The flora of the CON, MOD, and DMDD groups formed the Venn map. **(C)** Genus-level species composition analysis. LEfSe Analysis of **(D)** MOD, and DMDD. Main functional analysis of the **(E)** CON, MOD, and DMDD microflora.

The intestine and liver are closely linked through the gut-liver axis. An imbalance in the intestinal microbiota can disrupt the intestinal barrier function, allowing bacteria and their products from the intestine to translocate to the liver, leading to the occurrence and progression of liver diseases such as liver fibrosis. To determine whether DMDD affects the intestinal flora in liver fibrosis, we performed an intestinal flora analysis. The results showed that although the intestinal community structures among the CON, mod, and DMDD groups were similar, there was a trend of dispersion among the groups. The dispersion between the MOD and control groups was more obvious, and the DMDD and CON groups were relatively similar (Figure 4A; Supplementary Table S1). The analysis of common species of samples showed that there were 294 common species among the CON, MOD, and DMDD group (Figure 4B). Further species composition analysis showed that *Lactobacillus* and *Bacteroides* were significantly reduced and Ruminococcaceae_Ruminococcus and Oscillospira were significantly increased in the MOD group compared to the CON group at the genus level, while DMDD could improve this unbalanced relationship (Figure 4C).

To identify the microbiota biomarkers in each group, differences in the gut microbiome between the MOD and DMDD groups were evaluated by left analysis. At the genus level, the dominant genera in the MOD group were Ruminococcus and Bilophila, and the dominant genera in the DMDD group were Anaerotruncus, Parabacteroides, and Bilophila (Figure 4D). To further investigate the role of these differential gut microbes in liver fibrosis, we predicted the function of these differential microbes using piecrust. It was found that the differential flora was closely related to the biosynthesis of other secondary metabolites, glycan biosynthesis and metabolism, and metabolism of other amino acids (Figure 4E). In conclusion, there were differences in the composition of the intestinal flora among the CON, MOD, and DMDD groups. Although the composition of colonies in the DMDD and CON groups was different, it also shows that DMDD intervention can help the intestinal flora of mice with liver fibrosis develop to a healthy level.

### DMDD improves bile acid metabolism in mice with liver fibrosis

**FIGURE 5:**
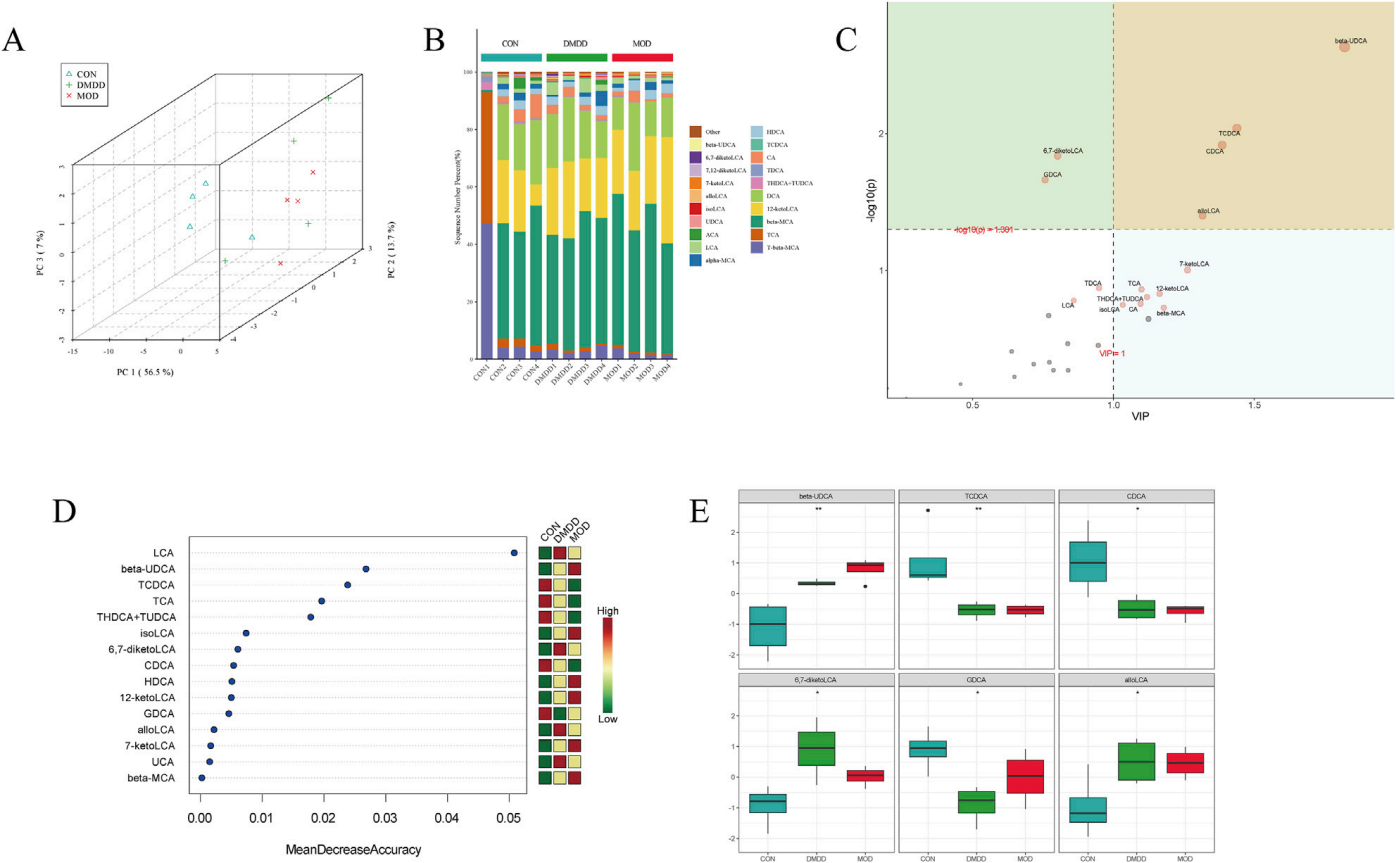
DMDD improves bile acid metabolism in CCL4-induced liver fibrosis in mice. **(A)** PCA of CON, MOD, and DMDD groups. Analysis of bile acid composition in the **(B)** CON, MOD, and DMDD groups. **(C)** CON, MOD, and DMDD Orthogonal Projections to Latent Structures Discriminant Analysis (OPLS-DA) Analysis. **(D)** Random deep forest plots of CON, MOD, and DMDD groups. **(E)** CON, MOD, and DMDD groups showed significant differences in bile acid levels. *P < 0.05, **P < 0.01,***P < 0.001,****P < 0.0001.

The metabolism of bile acids is closely linked to liver fibrosis, and an abnormal metabolism often indicates that the liver may undergo pathological changes. To understand the role of DMDD from the perspective of metabolism, this study used targeted metabolomics to analyze fecal metabolites. Principal component analysis (PCA) showed the effect of DMDD on bile acid metabolism, and the results showed that there was a large separation of bile acid structures in the CON, MOD, and DMDD groups (Figure 5A). Bile acid composition analysis showed that 12 kctolca, beta MCA, and TCA were the top three bile acids in each group (Figure 5B). We then used partial least squares discriminant analysis (PLS-DA) and the random forest method to select the characteristic bile acids with the largest difference and the highest contribution among the groups. The results showed that Taurochenodeoxycholic acid (TCDCA), Chenodeoxycholic acid (CDCA), allolca, β-ursodeoxycholic Acid (beta UDCA), and Lithocholic Acid (LCA) were the main differential bile acids (Figures 5C,D). Analysis of bile acid content showed that there were significant differences among the three groups in beta Ursodeoxycholic acid (UDCA), Taurochenodeoxycholic acid (TCDCA), CDCA, 6,7-diketolca, glycodeoxycholicacid (GDCA), and allolca. The level of beta UDCA in the feces of mice in the MOD group was significantly higher than that in the CON group, and the levels of TCDCA and CDCA were significantly lower than those in the CON group. These changes were partially improved after DMDD intervention (Figure 5E). In summary, these results showed that there were significant differences in bile acid metabolism between the CON and MOD groups and that DMDD intervention could change the bile acid metabolism of liver fibrosis in the CON group.

**FIGURE 6:**
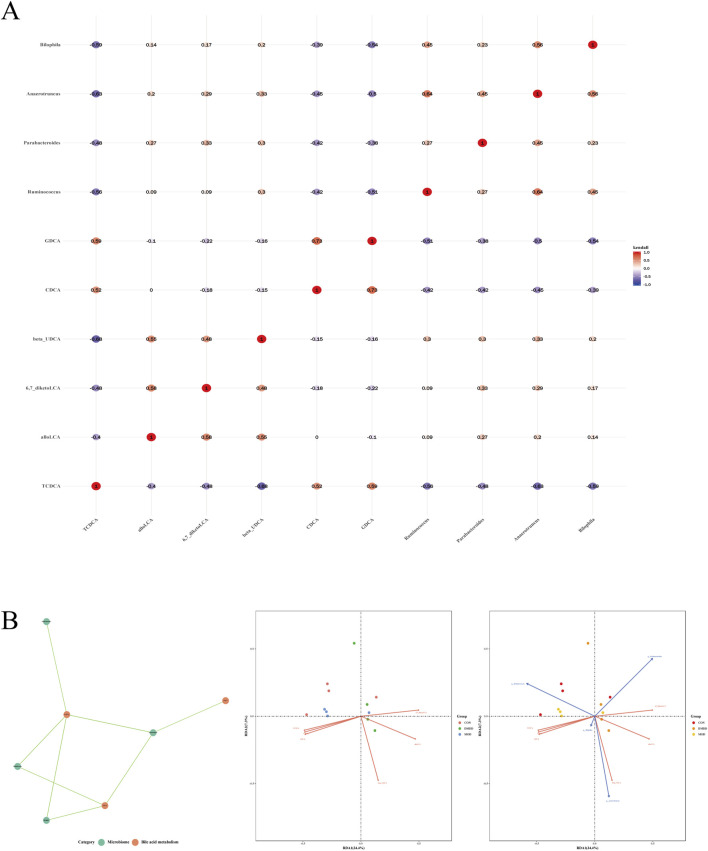
Correlation analysis between differential metabolic bile acids and flora. **(A)** differential metabolic bile acids are associated with bacterial flora. **(B)** correlation analysis of significantly different bile acids and flora.

To further evaluate the correlation between intestinal flora and bile acids, intestinal flora with LDA >4 and differentially metabolized bile acids were selected for analysis. The results showed that TCDCA was negatively correlated with *Ruminococcus*, *Parabacteroides*, *Anaerotruncus*, and *Bilophila*; CDCA was negatively correlated with *Anaerotruncus*; and GDCA was negatively correlated with *Ruminococcus*, *Anaerotruncus*, and *Bilophila* (Figures 6A,B). Correlation analysis between differential bile acids and samples showed that CDCA was positively correlated with the MOD group, while 6,7-diketolca, GDCA, and allolca were positively correlated with the DMDD group (Figure 6B).

## Discussion

At present, treatment for hepatitis virus and other etiologies has enabled some patients to obtain anti-fibrosis efficacy, but there is no relevant anti-fibrosis drug on the market (Xu and Liu, 2020). In recent years, an increasing number of scholars have found that traditional Chinese medicine has a multi-target comprehensive effect and has unique advantages and efficacy in inhibiting the progression of liver fibrosis and reversing liver fibrosis (Jiaorong et al., 2024; Yadong et al., 2024; Ying et al., 2024). Mining natural products with good anti-liver fibrosis properties from traditional Chinese medicine has become an important method for new drug research and development. As a medicinally valuable plant material, Averrhoa carambola root contains large amounts of vitamin C, various carbohydrates, organic acids, polyphenols, and flavonoids. Therefore, Averrhoa carambola roots have antioxidant and metabolic effects. To better understand the inhibitory effect of DMDD on liver fibrosis, we first analyzed the intestinal microbiota and bile acid metabolism. We found that DMDD helps maintain the balance of bile acid metabolism, thereby restoring the normal intestinal microbiota. Subsequently, we performed transcriptome sequencing of liver tissue and discovered that DMDD may exert its effects by inhibiting the TGF-β/Smad signaling pathway, which was initially confirmed in subsequent RT-PCR experiments. To our knowledge, this is the first study to elucidate the improvement in liver fibrosis from the perspective of the intestinal microbiota and bile acid metabolism, further confirming that DMDD may be a potential therapeutic drug for liver fibrosis.

Changes in the gut microbiota change with the progression of liver fibrosis, and dysbiosis of the gut microbiota can also aggravate liver fibrosis (Zhiming et al., 2020; Sujie et al., 2022). *Parabacteroides* is one of the core flora in the human intestine and mainly plays lipid-lowering, anti-inflammatory, and anti-tumor roles (Jessica et al., 2021). Studies have found that the levels of *Parabacteroides* in the feces of patients with clinical liver fibrosis and mouse liver fibrosis models are significantly reduced; therefore, it is presumed that *Parabacteroides* supplementation can resist the development of liver fibrosis (Foreman and Bouffet, 1999; Loomba et al., 2017). *Bilophila spp*. are opportunistic pathogens. The negative impact of its increased abundance on intestinal inflammation has been confirmed, and it is closely related to systemic inflammatory diseases and rectal cancer (Suzanne et al., 2012; Xuan et al., 2022). *Bilophila wadsworthia* is the most studied *Bilophila*, which synergizes with a high-fat diet to promote a higher inflammatory response, intestinal barrier dysfunction, and abnormal bile acid metabolism, resulting in higher abnormal glucose metabolism and hepatic lipogenesis (Keneko et al., 1993). *Ruminococcus* had a certain degree of heterogeneity. Some studies have shown that *Ruminococcus* is beneficial to health, but others have also shown that *Ruminococcus* promotes intestinal permeability and liver inflammation (Png et al., 2010; Sartor, 2011; Melissa et al., 2014). A study evaluating the association between gut microbiota dysbiosis and severe nonalcoholic fatty liver disease found that the accumulation of *Ruminococcus* was associated with the fibrosis stage (Jérôme et al., 2015). These studies showed that increasing beneficial flora could help improve liver fibrosis. Several studies have shown that curcumin, ursolic acid, and other traditional Chinese medicine monomers target the regulation of intestinal flora and metabolites, maintenance of intestinal microecological stability, inhibition of liver inflammation, and protection of hepatocytes, thereby improving liver fibrosis (Sizhe et al., 2020; Cheng et al., 2021; Ke et al., 2022; Yang et al., 2022). Therefore, this study explored the microbiome characteristics of mouse intestinal contents. After comparative analysis, we found that compared with the model group, DMDD significantly increased the abundance of *Anaerotruncus*, *Parabacteroides*, and *Bilophila* and significantly reduced the abundance of *Ruminococcus*. Our findings indicate that DMDD treatment has a positive effect on optimizing the intestinal flora structure in patients with liver fibrosis, which is expected to alleviate or even reverse the process of liver fibrosis.

To study changes in intestinal bile acids during the progression of liver fibrosis, we determined the bile acid profile in the intestine. The results showed that the bile acid profiles of the mice in each group were significantly different. The main finding was that the intestinal beta UDCA of mice in the liver fibrosis group was significantly increased, while TCDCA and CDCA were significantly decreased. This situation improved after DMDD treatment. β-UDCA can be isomerized by intestinal and liver enzymes to produce UDCA. UDCA has cholagogic, anti-apoptotic, anti-proliferative, and immune system-regulating effects, and has been widely used in the treatment of various chronic liver diseases and Parkinson’s disease (Gideon et al., 2018; Wanfu et al., 2022; Thomas et al., 2023). TCDCA is one of the main components of bile acids. It induces apoptosis through the TGR5 and PKC/JNK signaling pathways, regulates the expression of immune factors, and plays an important role in the immune response (Yuji et al., 2003; Xu et al., 2016). Recent research shows that TCDCA mediates the cAMP-PKA-CREB signaling pathway to exert anti-inflammatory and immunomodulatory effects (You-Chao et al., 2020). The current study shows that CDCA has a protective effect against pancreatitis and intestinal injury, contributing to the activation of intestinal stem cells, promoting the regeneration of intestinal epithelial cells, and regulating the composition of the intestinal immune cell population (Haojun et al., 2016; Baiqiang et al., 2018; Clarissa et al., 2020; Giovanni et al., 2020). We further analyzed the key genes involved in liver bile acid metabolism, and the results showed that DMDD upregulated the mRNA expression of CYP7A1, FXR, BSEP, and NTCP in liver tissue. These results suggest that DMDD alleviates bile acid accumulation and liver injury caused by liver fibrosis by maintaining the balance of bile acids in the intestinal hepatic axis.

Bile acids are closely related to the intestinal flora. After the primary conjugated bile acids enter the intestinal lumen, they are metabolized into secondary bile acids by intestinal flora. Secondary bile acids are reabsorbed in the terminal ileum and returned to the liver via the portal vein system to complete gut-liver axiscirculation (Sama et al., 2013; Annika et al., 2016). It can be seen that the change in the bile acid profile affects the abundance of intestinal flora, and the change of intestinal flora also leads to the change of the bile acid profile (Wei et al., 2017). Bile acids such as beta-UDCA, TCDCA, and CDCA play crucial regulatory roles in the gut-liver axis. They maintain the health of the intestine and liver by influencing bile acid metabolism, gut microbiota, and intestinal permeability. However, the specific mechanisms and effects of 6,7-diketoLCA, GDCA, and alloLCA within the gut-liver axis remain to be further explored. Therefore, it is necessary to strengthen research on the roles of these substances in the gut-liver axis in future studies to gain a more comprehensive understanding of their importance in maintaining human health (Wei et al., 2017). The data from this study showed that the differential bacteria were closely related to intestinal TCDCA, CDCA, and GDCA. We speculate that DMDD promotes the expression of bile acid transport genes in the liver by regulating the intestinal flora (especially *Ruminococcus* and *Parabacteroides*), thereby maintaining the balance of bile acid reabsorption and metabolism.

TGF-β is currently recognized as a profibrotic cytokine. When an injury occurs, TGF in the fibrotic environment- β, which stimulates downstream smad2/3 activation, targets downstream genes, and initiates the expression and secretion of extracellular matrix component genes (Erine et al., 2021). At present, a variety of TGFs have been developed as- β/ Inhibitors of Smad signal transduction, used to block fibrosis (Kelly et al., 2017; Andrea Hermina et al., 2018). Therefore, targeting the TGF-β/Smad signaling pathway may be a promising strategy to block or even reverse liver fibrosis. In the present study, after DMDD treatment, hepatic TGF-β, Smad2, and Smad3 were significantly downregulated, and COL1A1, α-SMA, and TIMP1 were key genes in liver fibrosis. It was preliminarily confirmed that DMDD inhibits TGF-β/Smad signal transduction, thereby playing an antifibrotic role. In summary, DMDD promotes the production of secondary bile acids (such as TCDCA) by modulating the gut microbiota (e.g., increasing *Parabacteroides*), which in turn activates FXR signaling in hepatocytes through the gut-liver axis, thereby inhibiting the TGF-β/Smad pathway.

**FIGURE 7:**
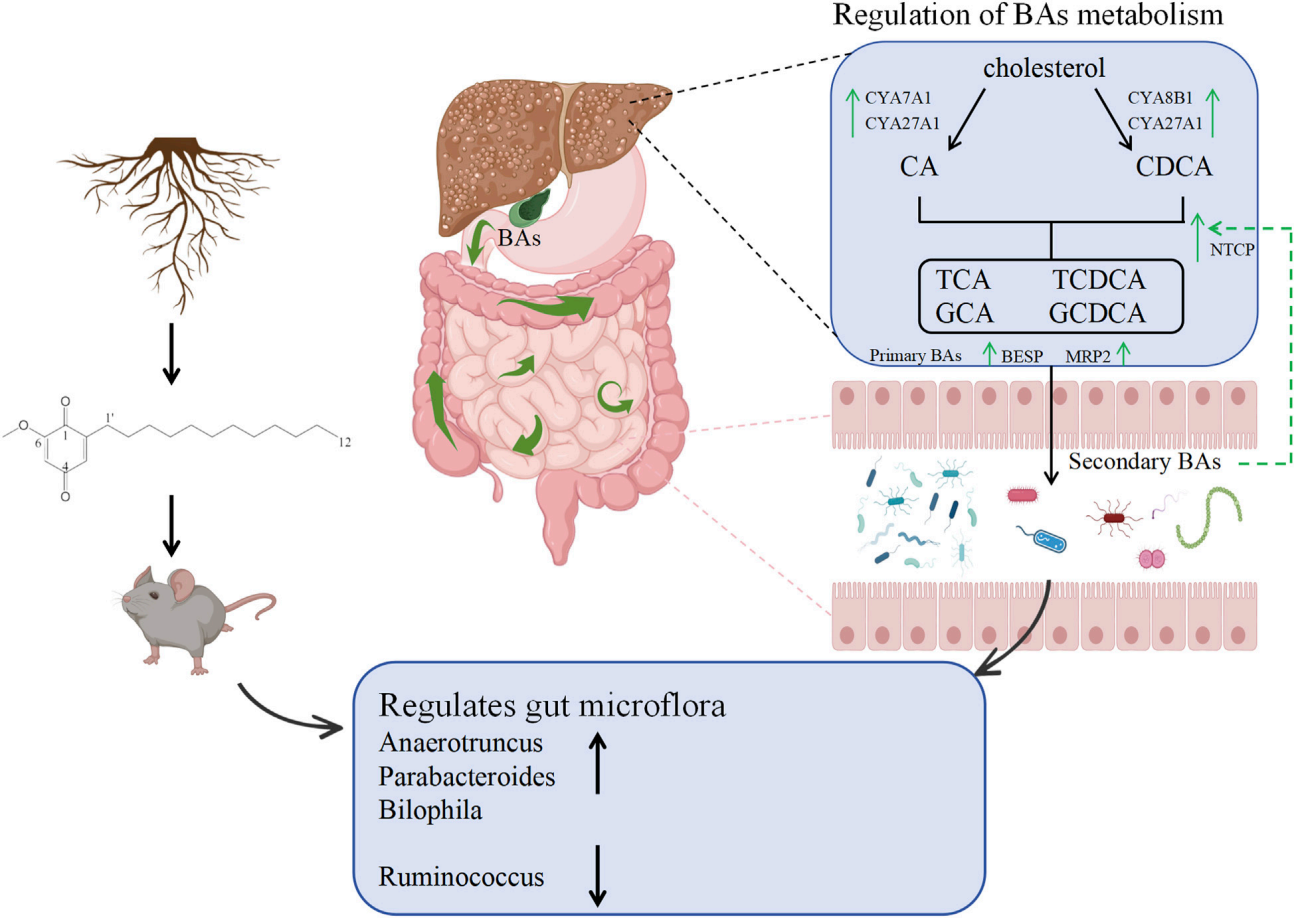
Mechanism diagram of DMDD regulating intestinal flora and bile acid metabolism in alleviating liver fibrosis.

DMDD can inhibit hepatic stellate cell activation and TGF-β pathway overactivation by up-regulating the expression of tight junction protein, reducing endotoxemia, and reducing enteric harmful substances entering the liver through the portal vein, thus ultimately alleviating fibrosis (Figure 7). This mechanism works synergically with the intestinal microbiota-bile acid metabolic axis to mediate the multi-target efficacy of DMDD. Our study bears certain limitations. Firstly, the number of mice used per group in this research is relatively small, albeit sufficient for preliminary exploratory experiments. Future studies will aim to increase the sample size to validate the robustness of our findings. Secondly, we did not opt for bile samples for bile acid sequencing. Given that the intestine serves as a crucial site for bile acid metabolism, intestinal samples offer a comprehensive reflection of the various bile acid components and their metabolic states. In contrast, while bile samples do contain bile acids, they may not fully capture the metabolic landscape of bile acids in the intestine due to challenges in sample collection and limitations in their representativeness.

## Conclusion

In summary, this study revealed that DMDD can regulate the TGF-β/Smad signaling pathway and repair the intestinal flora and bile acid metabolic balance to alleviate liver fibrosis. Therefore, DMDD may be a naturally active substance for the treatment of liver fibrosis.

## Data Availability

The transcriptome sequencing data presented in the study are deposited in the figshare repository, available at 10.6084/m9.figshare.28946027.

## Abbreviations

alloLCA, allolithocholic acid; LCA, Lithocholic Acid; isoLCA, isodeoxycholic acid; NorDCA, 23-nordeoxycholic acid; 7-ketoLCA, 7-ketodeoxycholic acid; 12-ketoLCA, 12-ketolithocholic acid; beta-UDCA, β-ursodeoxycholic Acid; DCA, Deoxycholic acid; CDCA, Chenodeoxycholic acid; UDCA, Ursodeoxycholic acid; HDCA, Hyodeoxycholic acid; NorCA, norcholic acid; 7,12-diketoLCA, 7,12-diketolithocholic acid; 6,7-diketoLCA, 6,7-Diketolithocholic acid; alpha-MCA, Tauro-α-muricholic acid; UCA, ursocholic acid; beta-MCA, β-muricholic acid; CA, Cholic acid; ACA, allocholic acid; GDCA, glycodeoxycholicacid; TDCA, Tauroursodeoxycholic acid; TCDCA, Taurochenodeoxycholic acid; TCA, Taurocholic acid.

## References

[B1] Ali M. Alan L. Saied S. Rafael L. Ali H. M. Jeff S. (2014). Liver cirrhosis mortality in 187 countries between 1980 and 2010: a systematic analysis. BMC Med. 12, 145. 10.1186/s12916-014-0145-y 25242656 PMC4169640

[B2] Andrea Hermina G. Alexandru-Emil M. Jörg H. W. D. (2018). Targeting TGF-β signaling for the treatment of fibrosis. Matrix Biol. 68-69, 8–27. 10.1016/j.matbio.2017.12.016 29355590

[B3] Annika W. Sama S. Hanns-Ulrich M. Fredrik B. (2016). Intestinal crosstalk between bile acids and microbiota and its impact on host metabolism. Cell Metab. 24, 41–50. 10.1016/j.cmet.2016.05.005 27320064

[B4] Asmaa A. Amal E.-K. Doaa A. Hoda M. K. Alyaa R. (2021). Modified lipid nanocapsules for targeted tanshinone IIA delivery in liver fibrosis. Int. J. Nanomedicine 16, 8013–8033. 10.2147/ijn.s331690 34916792 PMC8671377

[B5] Baiqiang L. Na Y. Chuling L. Chuwei L. Kun G. Xiaochun X. (2018). INT-777, a bile acid receptor agonist, extenuates pancreatic acinar cells necrosis in a mouse model of acute pancreatitis. Biochem. Biophys. Res. Commun. 503, 38–44. 10.1016/j.bbrc.2018.05.120 29859191

[B6] Cheng W. Cheng M. Ke F. Li-Hong G. Ya-Fang Z. Hong-Lin Z. (2021). Phillygenin attenuates carbon tetrachloride-induced liver fibrosis via modulating inflammation and gut microbiota. Front. Pharmacol. 12, 756924. 10.3389/fphar.2021.756924 34621179 PMC8490881

[B7] Chunxia C. Zhihuan N. Qiuqiao X. Junhui H. Wene C. Xiuneng T. (2017). 2-Dodecyl-6-methoxycyclohexa-2,5-diene-1,4-dione inhibits the growth and metastasis of breast carcinoma in mice. Sci. Rep. 7, 6704. 10.1038/s41598-017-07162-3 28751740 PMC5532290

[B8] Clarissa C. Peter M. Daniel K. Olga I. Michail S. Jacob V. (2020). Bacterial metabolism of bile acids promotes generation of peripheral regulatory T cells. Nature 581, 475–479. 10.1038/s41586-020-2193-0 32461639 PMC7540721

[B9] Erine B. Johanna S. Martin D. Scott T. Rik D. (2021). TGF-β as a driver of fibrosis: physiological roles and therapeutic opportunities. J. Pathol. 254, 358–373. 10.1002/path.5680 33834494

[B10] Foreman N. K. Bouffet E. (1999). Ependymomas in children. J. Neurosurg. 90, 605. 10.3171/jns.1999.90.3.0605 10067939

[B11] Fu-Sheng W. Jian-Gao F. Zheng Z. Bin G. Hong-Yang W. (2014). The global burden of liver disease: the major impact of China. Hepatology 60, 2099–2108. 10.1002/hep.27406 25164003 PMC4867229

[B12] Gideon M. H. Jessica D. Graeme J. M. A. Michael H. C. Jane C. Stefan H. (2018). The British Society of Gastroenterology/UK-PBC primary biliary cholangitis treatment and management guidelines. Gut 67, 1568–1594. 10.1136/gutjnl-2017-315259 29593060 PMC6109281

[B13] Ginès P. Krag A. Abraldes J. G. Solà E. Fabrellas N. Kamath P. S. (2021). Liver cirrhosis. Lancet 398 (10308), 1359–1376. 10.1016/s0140-6736(21)01374-x 34543610

[B14] Giovanni S. Alessia P. Ece Y. Gaby E. A. Maroun B. S. Antimo G. (2020). Bile acids signal via TGR5 to activate intestinal stem cells and epithelial regeneration. Gastroenterology 159, 956–968.e8. 10.1053/j.gastro.2020.05.067 32485177

[B15] Haojun Y. Haoming Z. Lin Z. Johan A. Kristina S. Xuehao W. (2016). Plasma membrane-bound G protein-coupled bile acid receptor attenuates liver ischemia/reperfusion injury via the inhibition of toll-like receptor 4 signaling in mice. Liver Transpl. 23, 63–74. 10.1002/lt.24628 27597295

[B16] Hongliang Z. Shunyu L. Lixiu C. Xiang H. Luhui J. Yuchun L. (2020). 2-Dodecyl-6-methoxycyclohexa-2,5-diene-1,4-dione, isolated from the root of Averrhoa carambola L., protects against diabetic kidney disease by inhibiting TLR4/TGFβ signaling pathway. Int. Immunopharmacol. 80, 106120. 10.1016/j.intimp.2019.106120 31972423

[B17] Jérôme B. Olaf M. Matthieu B. Mariana M. Lionel F. Felix A.-P. (2015). The severity of nonalcoholic fatty liver disease is associated with gut dysbiosis and shift in the metabolic function of the gut microbiota. Hepatology 63. 10.1002/hep.28356 PMC4975935 26600078

[B18] Jessica C. E. Daven S. Austin H. Hailey E. Daniel C. Sandra P. M. (2021). Parabacteroides distasonis: intriguing aerotolerant gut anaerobe with emerging antimicrobial resistance and pathogenic and probiotic roles in human health. Gut Microbes 13, 1922241. 10.1080/19490976.2021.1922241 34196581 PMC8253142

[B19] Jiaorong Q. Xiaoyong X. Zhixing W. Zhi M. Kexin J. Fanghong L. (2024). Si-Wu-Tang attenuates liver fibrosis via regulating lncRNA H19-dependent pathways involving cytoskeleton remodeling and ECM deposition. Chin. J. Nat. Med. 22, 31–46. 10.1016/s1875-5364(24)60560-1 38278557

[B20] Ke F. Cheng M. Cheng W. Honglin Z. Lihong G. Yafang Z. (2022). Forsythiaside A alleviated carbon tetrachloride-induced liver fibrosis by modulating gut microbiota composition to increase short-chain fatty acids and restoring bile acids metabolism disorder. Biomed. Pharmacother. 151, 113185. 10.1016/j.biopha.2022.113185 35623173

[B21] Kelly L. W. Katharine J. Craig H. (2017). Targeting TGF-β mediated SMAD signaling for the prevention of fibrosis. Front. Pharmacol. 8, 461. 10.3389/fphar.2017.00461 28769795 PMC5509761

[B22] Keneko H. Nakanishi Y. Taya K. Kishi H. Watanabe G. Sasamoto S. (1993). Evidence that inhibin is an important factor in the regulation of FSH secretion during the mid-luteal phase in cows. J. Endocrinol. 136, 35–41. 10.1677/joe.0.1360035 8429275

[B23] Loomba R. Seguritan V. Li W. Long T. Klitgord N. Bhatt A. (2017). Gut microbiome-based metagenomic signature for non-invasive detection of advanced fibrosis in human nonalcoholic fatty liver disease. Cell Metab. 25 (5), 1054–1062.e5. 10.1016/j.cmet.2017.04.001 28467925 PMC5502730

[B24] Maurizio P. Massimo P. (2023). Liver fibrosis in NAFLD/NASH: from pathophysiology towards diagnostic and therapeutic strategies. Mol. Asp. Med. 95. 10.1016/j.mam.2023.101231 38056058

[B25] Melissa R. C. John D. David S. Andrew C. Shanti B. Paul J. W. (2014). Unique aspects of fiber degradation by the ruminal ethanologen Ruminococcus albus 7 revealed by physiological and transcriptomic analysis. BMC Genomics 15, 1066. 10.1186/1471-2164-15-1066 25477200 PMC4300822

[B26] Png C. W. Sara L. Kristen S. G. Erwin Z. Chris S. M. Lindsay I. S. (2010). Mucolytic bacteria with increased prevalence in IBD mucosa augment *in vitro* utilization of mucin by other bacteria. Am. J. Gastroenterol. 105, 2420–2428. 10.1038/ajg.2010.281 20648002

[B27] Sama S. Annika W. Jenny F. Sirkku J. Hanns-Ulrich M. Krister B. (2013). Gut microbiota regulates bile acid metabolism by reducing the levels of tauro-beta-muricholic acid, a naturally occurring FXR antagonist. Cell Metab. 17, 225–235. 10.1016/j.cmet.2013.01.003 23395169

[B28] Sartor R. B. (2011). Key questions to guide a better understanding of host-commensal microbiota interactions in intestinal inflammation. Mucosal Immunol. 4, 127–132. 10.1038/mi.2010.87 21248723

[B29] Sizhe W. Chenkai H. Anjiang W. Xuan Z. (2020). Ursolic acid improves the bacterial community mapping of the intestinal tract in liver fibrosis mice. PeerJ 8, e9050. 10.7717/peerj.9050 32355580 PMC7185030

[B30] Sujie L. Juan W. Pingping C. Shadi A. D. M. Jingbo Z. Shumin L. (2022). TAK-242 ameliorates hepatic fibrosis by regulating the liver-gut Axis. Biomed. Res. Int. 2022, 4949148. 10.1155/2022/4949148 36017390 PMC9398794

[B31] Suzanne D. Yunwei W. Mark M. Vanessa L. Hannah F.-P. Anuradha N. (2012). Dietary-fat-induced taurocholic acid promotes pathobiont expansion and colitis in Il10-/- mice. Nature 487, 104–108. 10.1038/nature11225 22722865 PMC3393783

[B32] Thomas P. Matthew A. Ellen B. Linda M. A. Benjamin H. M. Matilde S. (2023). A double-blind, randomized, placebo-controlled trial of ursodeoxycholic acid (UDCA) in Parkinson’s disease. Mov. Disord. 38, 1493–1502. 10.1002/mds.29450 37246815 PMC10527073

[B33] Tiasha D. Venkatraman M. (2023). Fibrosis in liver and pancreas: a review on pathogenic significance, diagnostic options, and current management strategies. Inflammation 46, 824–834. 10.1007/s10753-022-01776-0 36595108

[B34] Wanfu L. Shu L. Yongbin M. Guokai H. Shufang L. Juan D. (2022). UDCA inhibits hypoxic hepatocellular carcinoma cell-induced angiogenesis through suppressing HIF-1α/VEGF/IL-8 intercellular signaling. Front. Pharmacol. 12. 10.3389/fphar.2021.755394 PMC8714963 34975472

[B35] Wei J. Guoxiang X. Weiping J. (2017). Bile acid-microbiota crosstalk in gastrointestinal inflammation and carcinogenesis. Nat. Rev. Gastroenterol. Hepatol. 15, 111–128. 10.1038/nrgastro.2017.119 29018272 PMC5899973

[B36] Xu L. M. Liu P. Hepatology Committee of Chinese Association of Integrative Medicine, China (2020). Guidelines for diagnosis and treatment of hepatic fibrosis with integrated traditional Chinese and Western medicine (2019 edition). J. Integr. Med. 18 (3), 203–213. 10.1016/j.joim.2020.03.001 32331978

[B37] Xu W. Ziying Z. Xiuling H. Wei M. Lei Z. Peifeng L. (2016). Taurochenodeoxycholic acid induces NR8383 cells apoptosis via PKC/JNK-dependent pathway. Eur. J. Pharmacol. 786, 109–115. 10.1016/j.ejphar.2016.06.007 27268718

[B38] Xuan Z. Lin J. Xiuyu F. Zhiqiang G. Xiaoxu W. Baoming S. (2022). Host-microbiota interaction-mediated resistance to inflammatory bowel disease in pigs. Microbiome 10, 115. 10.1186/s40168-022-01303-1 35907917 PMC9338544

[B39] Yadong F. Xiaoxi Z. Lin W. Weiguo F. Siqi G. Danyan Z. (2024). Salvianolic acid B attenuates liver fibrosis by targeting Ecm1 and inhibiting hepatocyte ferroptosis. Redox Biol. 69, 103029. 10.1016/j.redox.2024.103029 38184998 PMC10808927

[B40] Yang Z. Jiahui W. Jiaru W. Ruizhu J. Tiejian Z. (2022). Gut microbiota combined with metabolomics reveal the mechanism of curcumol on liver fibrosis in mice. Biomed. Pharmacother. 152, 113204. 10.1016/j.biopha.2022.113204 35653891

[B41] Yanwei L. Yunrui L. Mozuo N. Qiuju S. Chong Z. Chao H. (2023). Therapeutic potential and mechanism of Chinese herbal medicines in treating fibrotic liver disease. Chin. J. Nat. Med. 21, 643–657. 10.1016/s1875-5364(23)60443-1 37777315

[B42] Ying M. Yuancheng B. Han W. Huaizhou J. Lei Z. Bo Y. (2024). 1H-NMR-based metabolomics to dissect the traditional Chinese medicine promotes mesenchymal stem cell homing as intervention in liver fibrosis in mouse model of Wilson's disease. J. Pharm. Pharmacol. 76, 656–671. 10.1093/jpp/rgae016 38429940

[B43] Yongfei H. Yanghong L. Tianyi L. Shutian M. Yuan L. Zijun C. (2022). Effects of 2-dodecyl-6-methoxycyclohexa-2,5-diene-1,4-dione on autophagy and the PI3K/AKT/mTOR signaling pathway in human cholangiocarcinoma QBC939 cells. J. Gastrointest. Oncol. 13, 1423–1432. 10.21037/jgo-22-298 35837172 PMC9274070

[B44] Yongxiang S. Yajun H. Guorong Y. Xuyou L. Jiahuang H. Qinghui Z. (2023). Curcumin inhibits the activity and induces apoptosis of activated hepatic stellate cell by suppressing autophagy. J. Cell Biochem. 124, 1764–1778. 10.1002/jcb.30487 37909649

[B45] You-Chao Q. Guo-Zhen D. Wei M. Qian L. Yong-Liang Z. Pei-Feng L. (2020). Taurochenodeoxycholic acid mediates cAMP-PKA-CREB signaling pathway. Chin. J. Nat. Med. 18, 898–906. 10.1016/s1875-5364(20)60033-4 33357720

[B46] Yuji K. Ryo F. Masaki H. Masataka H. Hiromi Y. Masanori M. (2003). A G protein-coupled receptor responsive to bile acids. J. Biol. Chem. 278, 9435–9440. 10.1074/jbc.M209706200 12524422

[B47] Zhiming L. Ming N. Haiyang Y. Lili W. Xiaoming Z. Tao C. (2020). Gut microbiota and liver fibrosis: one potential biomarker for predicting liver fibrosis. Biomed. Res. Int. 2020, 3905130. 10.1155/2020/3905130 32685479 PMC7322594

