# Supplementary figures and images for: 2-Dodecyl-6-methoxycyclohexa-2,5-dien-1,4-dione alleviates liver fibrosis and improves intestinal flora and bile acid metabolism
Source: Front Pharmacol. 2025 May 16;16:1581138. doi: 10.3389/fphar.2025.1581138 (PMC12122303; doi:10.3389/fphar.2025.1581138)

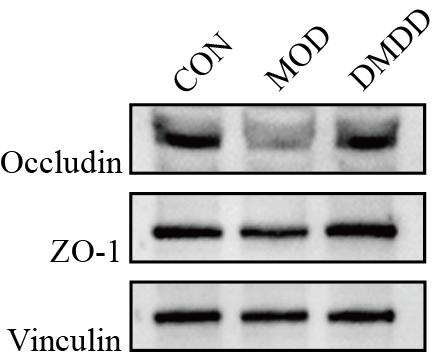

Supplement: Supplementary file 2 [file Image2.tif]

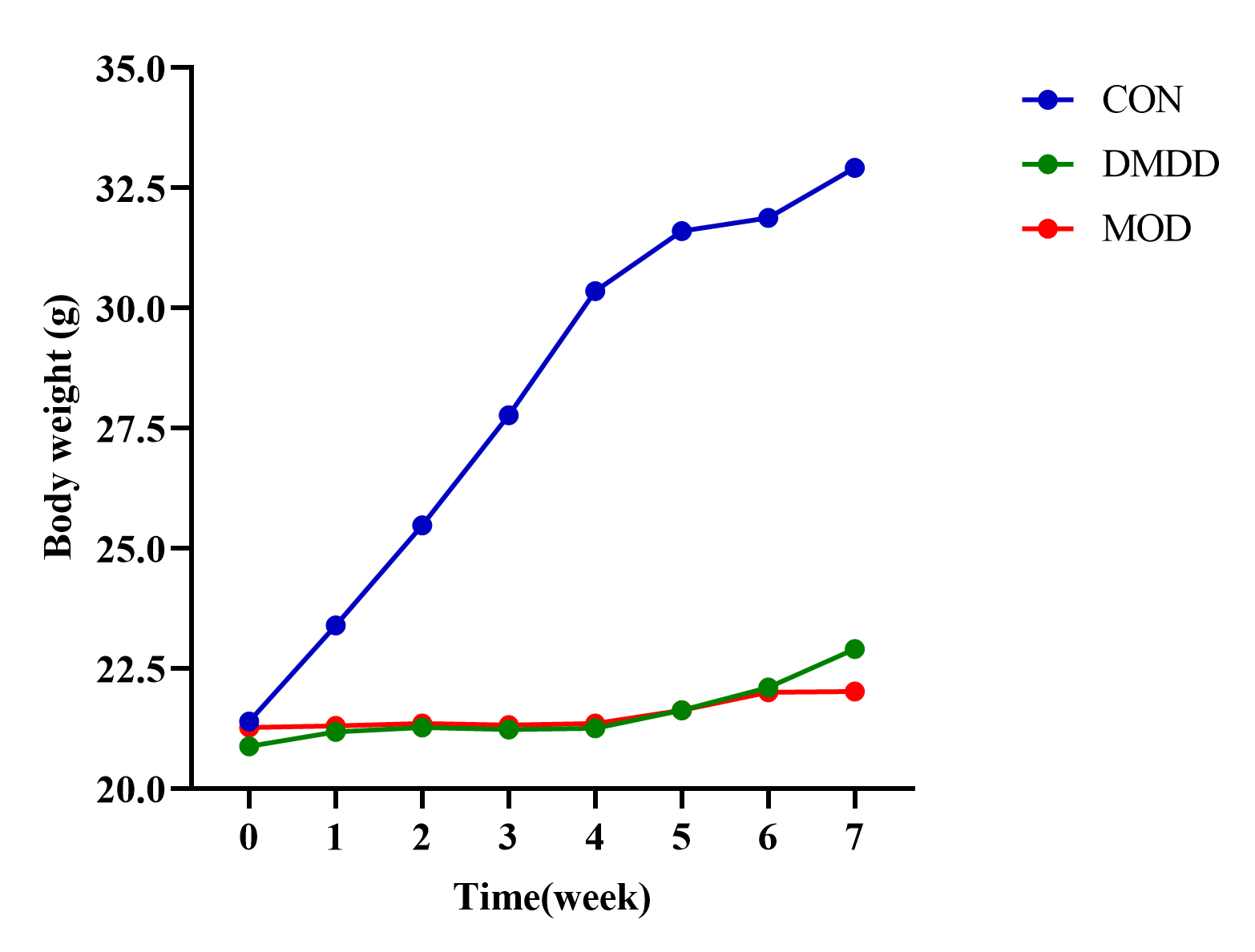

Supplement: Supplementary file 3 [file Image1.tif]
